# Effect of cyanide-utilizing bacteria and sulfur on feed utilization, microbiomes, and cyanide degradation in cattle supplemented with fresh cassava root

**DOI:** 10.1038/s41598-023-45993-5

**Published:** 2023-10-31

**Authors:** Napudsawun Sombuddee, Rittikeard Prachumchai, Waroon Khota, Waewaree Boontiam, Anusorn Cherdthong

**Affiliations:** 1 Department of Animal Science, Faculty of Agriculture, Tropical Feed Resources Research and Development Center (TROFREC), Khon Kaen University, Khon Kaen, 40002 Thailand; 2 Department of Animal Science, Faculty of Agricultural Technology, Rajamangala University of Technology Thanyaburi, Pathum Thani, 12130 Thailand; 3 Department of Animal Science, Faculty of Natural Resources, Rajamangala University of Technology Isan, Sakon Nakhon, 47160 Thailand

**Keywords:** Biochemistry, Biological techniques, Biotechnology, Microbiology, Molecular biology, Zoology

## Abstract

This study aimed to compare the effects of adding cyanide-utilizing bacteria (CUB) and sulfur on rumen fermentation, the degradation efficiency of hydrogen cyanide (HCN), feed utilization, and blood metabolites in beef cattle fed two levels of fresh cassava root (CR). A 2 × 2 factorial arrangement in a 4 × 4 Latin square design was used to distribute four male purebred Thai native beef cattle (2.5–3.0 years old) with an initial body weight (BW) of 235 ± 15.0 kg. Factor A was *Enterococcus faecium* KKU-BF7 oral direct fed at 10^8^ CFU/ml and 3% dry matter (DM) basis of pure sulfur in concentrate diet. Factor B was the two levels of CR containing HCN at 300 and 600 mg/kg on DM basis. There was no interaction effect between CUB and sulfur supplementation with CR on feed utilization (*p* > 0.05). Similarly, CUB and sulfur supplementation did not affect (*p* > 0.05) DM intake and apparent nutrient digestibility. However, the high level of CR supplementation increased (*p* < 0.05) feed intake and neutral detergent fiber digestibility. The ruminal pH, microbial population, ammonia–nitrogen, blood urea nitrogen, and blood thiocyanate concentrations were unaffected by the addition of CUB and sulfur at two CR concentrations (*p* > 0.05). The addition of CUB or sulfur had no effect on the efficiency of HCN degradation in the rumen (*p* > 0.05). However, cattle given CR with HCN at 600 mg/kg DM had considerably higher degradation efficiency than those fed CR containing HCN at 300 mg/kg DM (p < 0.05). The group fed CUB had a considerably greater CUB population (*p* < 0.05) than the sulfur group. Cyanide-utilizing bacteria or sulfur supplementation with CR had no interaction effect between total VFAs and their profiles (*p* > 0.05). However, the study observed a significant positive correlation between the amount of CR and the concentration of propionate in the rumen (*p* < 0.05). The levels of nitrogen absorption and nitrogen retention did not differ significantly among the treatments (p > 0.05). Hence, it may be inferred that the administration of a high concentration of CR at a dosage of 600 mg/kg DM HCN could potentially provide advantageous outcomes when animals are subjected to oral CUB incorporation.

## Introduction

Cassava is a crop that was traditionally produced in the tropics and subtropics, particularly in the northeastern region of Thailand, where it was used for both human food and animal feed^1^. The use of cassava chips as a source of energy for the production of ruminants in tropical nations has recently piqued the curiosity of the scientific community. However, there is still a need for and growing interest in substitute feed ingredients for cattle feeding due to the high cost of cassava chips and competition from the human food and biofuel sectors^1,2^. Fresh cassava root (CR) is a desirable option as the primary carbohydrate supplement in cow diets since it is less expensive than cassava chips. However, because it contains high quantities of hydrogen cyanide (HCN), which causes poisoning symptoms, raw cassava root is not recommended for animal feeding^3,4^. The findings of a previous study indicated that animal feed ingredients with a HCN content over 200 mg/kg DM pose a significant risk to animals^5,6^. As HCN is released in the rumen, it is taken into the bloodstream. Impermeable complexes between free HCN and oxidized iron (Fe^3+^) develop in cytochrome complexes^7^. Due to the inhibition of mitochondrial electron transport, the affected animals experience anoxia and death^8^. Hydrogen cyanide is swiftly absorbed in large amounts because it is so toxic and transcends the body’s detoxification mechanisms that death occurs in less than 2 h^9^.

When ruminants were fed HCN sources such as cassava root, extra elemental sulfur, according to a prior study, could lower HCN^6,10,11^. It was believed that sulfur served as a source of substrate for the rumen microbial cells to produce the rhodanese enzyme^12,13^. According to Supapong et al.^9^ after 7 days of ensilage, 2% sulfur added to a fermented total mixed meal containing 40% CR can reduce HCN by 37%. Feeding sulfur-containing feed blocks up to 40 g/kg with CR up to 15 g/kg of body weight had no detrimental effects on animal health^6^. Although ruminant HCN levels may be decreased by feeding CR with sulfur, the chemical process of HCN detoxification takes into account environmental fluctuations, residue, operational risks, and the creation of additional harmful compounds for consumers^14^. One of the most fascinating biological topics is the use of microbes to minimize HCN in animal feed. Increasingly than 35 different species of microorganisms have been identified as cyanide predators from a variety of sources, including soil, wastewater, sludge, and plants^15^. Microorganisms and HCN ingestion in non-rumen habits are becoming more common^16^. Cyanide-utilizing bacteria, also known as CUB, were first isolated from the rumen of ruminant animals in order to assess the efficiency with which these bacteria degraded HCN^17^. In an earlier study conducted by Sombuddee et al.^18^, the in vitro degradation efficiency (DE) of HCN was measured when CUB was administered at 10^8^ CFU/ml. The researchers found that the DE of HCN increased to 54.33% unit when 600 mg/kg DM HCN was used in the in vitro experiment^19^. However, HCN reduction by the CUB and sulfur has not yet been confirmed in vivo. It was hypothesized that the addition of CUB and sulfur would lower the HCN content in the animals ingesting CR. This study aimed to examine the effects of cyanide-utilizing bacteria and sulfur addition on rumen fermentation, degradation efficiency of HCN, feed utilization, and blood metabolites in beef cattle fed CR.

## Materials and methods

Experimental research on plants, including the collection of plant material, complied with relevant institutional, national, and international guidelines and legislation. We had the appropriate permissions/licenses to perform the experiment in the study area. The Animal Ethics Committee of Khon Kaen University, National Research Council of Thailand, approved all of the experimental animals and methodology used in this study (record no. IACUC-KKU-45/2564). During the course of the investigation, neither anesthesia nor animal sacrifice was performed. Our research confirmed that all methods were carried out in accordance with the applicable guidelines and regulations. The research was done in accordance with the ARRIVE guidelines.

### Cattle, treatments, experimental design, and feeding

A 2 × 2 factorial arrangement in a 4 × 4 Latin square design was used to distribute four male purebred Thai native beef cattle (2.0–3.0 years old) with an initial body weight (BW) of 235 ± 15.0 kg. Factor A was *Enterococcus faecium* KKU-BF7; *E. faecium* KKU-BF7 (10^8^ CFU/ml) and 3% dry matter of pure sulfur. Factor B was the two levels of fresh cassava root (CR) containing HCN at 300 and 600 mg/kg on DM basis. The novel cyanide-utilizing bacteria (*E. faecium* KKU-BF7) was previously isolated from swamp buffalo rumen fluids and was chosen based on their rhodanese activities^17^. Fresh cassava root (*Manihot esculenta* Kasetsart 50) was purchased from local farmers in Thailand’s Khon Kaen Province. Before being fed to the animals, the CR was cleaned to eliminate dirt and sliced into 3 to 5 mm-sized pieces. First, the animals underwent testing before the trial started for 14 days, and they were offered a CR intake at HCN present at 300 and 600 mg/kg DM, respectively, which they completely consumed. Daily, at 7:30 a.m. and 15:30 p.m., concentrate and CR were fed*. E. faecium* KKU-BF7 (10^8^ CFU/ml) was orally direct-fed every 7 days. *E. faecium* KKU-BF7 was cultured for 24 h in Lactobacilli MRS Broth medium (Difco Laboratories, Detroit, MI, USA) and absorbance at 660 nm was measured, obtaining approximately 10^8^ CFU/ml^16^. Rice straw was fed ad libitum, while concentrate was fed at 1% BW. Each feeding day's intake and the residual feed (5–10%) were noted. Each animal had access to clean, fresh-water while being housed in a 3 × 4 m cage. There were four feeding periods lasting 112 days (28 days per period). Each session comprises a duration of 21 days, during which 14 days are designated for feed adaptation. Subsequently, the animals are transferred to the metabolism crate for the remaining 7 days, during which assessments are conducted to evaluate digestibility, rumen parameters, and blood metabolites. Before commencing the subsequent phase, all animals were exclusively fed a basal diet for a duration of 7 days to facilitate rumen wash purposes, without the incorporation of any cassava supplementation. The total volume of urine and feces samples was collected daily, and a representative 5% of each sample used for later analysis. Table 1 lists the components and chemical composition of the concentrate feed that was fed to steers. Concentrate diets contained 88.82–89.60%, 14.39–15.13%, 27.37–29.63%, and 6.98–7.71% DM of DM, CP, NDF, and ADF, respectively. In the experimental diets, corn meal served as the main energy source, while protein sources included urea, soybean meal, palm kernel meal, and sulfur supplemented at 0% and 3%. The CR consisted of DM, CP, NDF, ADF, and HCN concentrations at 38.50%, 2.30%, 15.60%, 8.90% DM, and 262.08 mg/kg DM, respectively. The roughage source used was rice straw, which had high NDF and ADF fiber levels of 65.30% and 55.20% DM, respectively.

**Table 1:** Chemical composition of the experiment diet.

Item	Concentrate diet		Fresh cassava root (CR)	Rice straw
	0% Sulfur	3% Sulfur		
Ingredients, % dry matter (DM)				
Soybean meal (SBM)	10	10		
Palm kernel meal	12	12		
Rice bran	13	10		
Corn	60	60		
Molasses	2	2		
Salt	1	1		
Urea	1	1		
Mineral and vitamins^1^	1	1		
Sulfur powder	0	3		
Chemical composition				
Dry matter, %	89.60	88.82	38.50	97.20
Organic matter, %DM	95.93	95.39	92.40	90.10
Crude protein, %DM	15.13	14.39	2.30	2.60
Ether extract, %DM	2.65	2.46	1.21	2.01
Neutral detergent fiber, %DM	29.63	27.37	15.60	65.30
Acid detergent fiber, %DM	7.71	6.98	8.90	55.20
Non-fiber carbohydrate, %DM	48.52	51.17	73.29	20.19
Hydrocyanic acid, mg/kg DM	–	–	262.08	–

^1^Contains per kilogram premix: 10,000,000 IU vitamin A; 70,000 IU vitamin E; 1,600,000 IU vitamin D; 50 g iron; 40 g zinc; 40 g manganese; 0.1 g cobalt; 10 g copper; 0.1 g selenium; 0.5 g iodine.

### Measurement and chemical analysis

During the final 7 days of each period, data on the feed offered and the refusal of rice straw, CR, and concentrate were recorded. The total collection method was used to measure and sample daily amounts of feces and urine during the final 7 days of each session when the animals were housed in metabolic crates in order to evaluate feed digestion and nitrogen metabolism. The metabolic crates were equipped with robust slatted concrete floors, each featuring a trapezoidal stainless-steel sheet positioned beneath it. This stainless-steel sheet served as a conduit for the urine, directing it through a stainless-steel funnel into a plastic container. To prevent contamination with feces, this container was securely covered with a clean filter cloth. Within each crate, a urine tray was centrally placed, while a feces tray was situated at the rear. This arrangement facilitated the easy collection and separation of the animals’ urine and feces. Every day for the previous 7 days, 5% of the total fresh weight of the fecal samples was subsampled and divided into two parts: one part was used for the daily DM analysis, and another part were stored in the refrigerator and pooled by steer at the end of each period for chemical analysis. Bottles were used to collect urine, which was then preserved with 10% H_2_SO_4_ (v/v).

Samples of feed, refusals, and feces were subjected to drying in an oven preset to a temperature until achieving a constant weight of 60 °C. After that, samples were ground (1 mm screen using the Cyclotech Mill, Tecator, Hoganas, Sweden) to determine their nutritional content. The chemical composition of the samples was evaluated for DM (ID 967.03), ash (ID 942.05), ether extract (EE; ID 954.02) and crude protein (CP; ID 984.13) using the AOAC method^20^. The amount of neutral detergent fiber (NDF) and acid detergent fiber (ADF) in samples was evaluated using Van Soest et al.^21^, with the addition of alpha-amylase but without the addition of sodium sulfite. Non-fiber carbohydrate (NFC) was calculated according to the equation^22^: NFC (%) = 100 − (CP + NDF + EE + ash).

The level of HCN in feeds was measured by modifying Fisher and Brown’s^23^ picric acid method. After 10 min of centrifugation at 15,000×*g* at 4 °C, 0.1 ml aliquots of a 0.5% (w/v) picric acid solution and 0.25 M Na_2_CO_3_ were added to 0.05 ml aliquots of standard KCN solutions to create a linear calibration curve. The resulting combinations were then heated for 5 min, diluted to 1 ml with 0.85 ml of distilled water, and chilled in tap water for 30 min. Using a spectrophotometer and a blank of distilled water and a picric acid reagent, the absorbance at 520 nm was determined.

Urine samples were tested for urine-N using the Kjeldahl methodology^20^ and computed for N absorption (g): N intake (g)—N faecal (g), and N retention (g): N absorption (g)—N urine (g).

Blood samples were collected from the jugular vein using a 21-gauge needle at two-time points, 0 h and 4 h after eating, over each 21-day period. The purpose of collecting these 10 ml samples was to identify blood metabolites. Blood samples were drawn into tubes containing 12 mg of the anticoagulant Ethylenediaminetetraacetic acid (EDTA), and the plasma was isolated using centrifugation at a speed of 500 g for a duration of 10 min at a temperature of 4 °C. Later, blood urea nitrogen (BUN) (L type Wako UN, Tokyo, Japan) and blood thiocyanate measurements were made with a spectrophotometry (UV/VIS Spectrometer, PG Instruments Ltd., London, UK)^24^.

Concurrently with the collection of blood samples, a volume of about 100 ml of rumen fluid was obtained (at 0 h and 4 h after feeding) from the central region of the rumen using a stomach tube bound to a vacuum pump. In any case, the samples were collected by skilled collectors, who threw away the fluid if there was saliva in it to avoid interfering with the measurement of pH. The samples were immediately examined for pH using a pH meter (HANNA Instruments HI 8424 microcomputer, Kallang, Singapore). Rumen fluid samples were subjected to filtration using a four-layer cheesecloth. The samples were partitioned into four aliquots. One aliquot was utilized for the determination of ammonia–nitrogen (NH_3_–N) and volatile fatty acid (VFA). This was achieved by employing a plastic container containing 5 ml of 1 M H_2_SO_4_, to which 45 ml of rumen fluid was added. Spectrophotometry (UV/VIS Spectrometer, PG Instruments Ltd., London, UK) was used to measure the amount of NH_3_N. Total volatile fatty acids, acetate, propionate, and butyrate concentrations were measured using gas chromatography (Wilmington, DE 5890A Series II gas chromatograph and a glass column (180 cm, 4 mm) packed with 100 g/l SP-1200/10 g/l H_3_PO_4_ on 80/100 mesh Chromosorb WAW; Supelco, Bellefonte, PA, USA). The samples were centrifuged at 16,000 × *g* for 15 min. The concentration of HCN in rumen fluids was measured using the same methodology described above, and another part of the sample was stored in a 10% formalin solution (1:9 ratio) for bacterial protozoa and fungi study using a hemocytometer (Boeco, Hamburg, Germany)^25^. The cyanide-based bacteria present in the final portion of rumen fluid were examined using real-time PCR techniques^26^. The primer sets were created using the *Enterococcus faecium* (*E. faecium*) strain KKU-BF7 sequence and Primer3 software^27^. The sequences of the primers were: forward primer: CCATGTGTAGCGGTG AAATG and reverse primer: CGGAAACCCTCCAACACTTA. The DNA extraction was performed using the QIAamp DNA Mini kit (Qiagen), following a method for bacterial cells described in the manufacturer’s instructions^28^. For a reliable real-time PCR test, the template DNA isolated from *Enterococcus faecium* was normalized to 2 ng/l with deionized and distilled water (DDW). Using a LightCycler instrument and version 3.5 software, real-time PCR amplification and analysis were carried out (Roche Diagnostics, Rotkreuz, Switzerland)^29^. The thermal cycling protocol consisted of 40 cycles of 10 s each at 95 °C, 10 s at 62 °C, and 10 s at 72 °C. The initial denaturation took place for 10 min at 95 °C. The fluorescence signal was measured at the end of each extension step at 72 °C. After the amplification, it was confirmed that only the designated products were amplified by performing a melting curve analysis with a temperature gradient of 0.1 °C/s from 70 to 95 °C^30^.

### Statistical analysis and calculations

Variances were analyzed according to a 2 × 2 factorial arrangement in a 4 × 4 Latin square design using the GLM procedure of SAS version 9.00 (SAS Institute Inc., Cary, NC, USA). The model tested the random effects of beef cattle and period and the fixed effects of treatment. The data was analyzed using the model:

$${\text{Y}}_{{{\text{ijk}}}} = + {\text{ M}}_{{\text{i}}} + {\text{ E}}_{{\text{j}}} + {\text{ A}}_{{\text{k}}} + {\text{ P}}_{{\text{l}}} + {\text{ ME}}_{{{\text{ij}}}} + \, \varepsilon_{{{\text{ijk}}}} ,$$

where Y_ijk_ is the variance, μ is the overall mean, M_i_ is the CUB and sulfur (i = 1, 2), E_j_ is levels of CR containing HCN supplemented at 300 mg/kg DM and 600 mg/kg DM (i = 1, 2), A_k_ is the effect of the ruminant (k = 1, 2, 3, 4); P_l_ is the effect of the each period (l = 1, 2, 3, 4); ME_ij_ is the interaction effect, and ε_ijk_ is the residue. The means of the variances were reported with the standard error of the mean. Tukey’s multiple comparison test was run to check the statistical differences in treatment means at *p* < 0.05.

## Results

### Feed consumption and digestibility

Table 2 shows the results of CUB and sulfur supplemented with CR containing HCN at 300 mg/kg DM and 600 mg/kg DM. There was no interaction impact between CUB and sulfur supplementation with CR on the feed intake, nutrient intake, or nutrient digestibility of fresh cassava, condensed feed, or rice straw (*p* > 0.05). However, the level of CR supplementation affected the total feed intake of Thai native beef cattle. The total intake ranged from 6.95–8.09 kg/day to 105.62–123.47 g/kg BW^0.75^ (*p* < 0.01). There was a substantial increase in non-fiber carbohydrates (NFC) observed in animals that were fed a CR containing HCN at a concentration of 600 mg/kg DM (*p* < 0.01). The group receiving CR containing 600 mg/kg DM of HCN had an NFC intake higher by 0.82 kg/day in comparison to the group supplemented with 300 mg/kg DM of HCN in CR. The digestibility of NDF varied with the amount of CR fed, with the group receiving 600 mg/kg DM HCN having the highest levels (*p* < 0.05).

**Table 2:** Feed intake and digestibility of Thai native beef cattle fed two levels of fresh cassava root (CR) with cyanide-utilizing bacteria and sulfur.

Items	*Enterococcus faecium* (10^8^ CFU/ml)		3% Sulfur		SEM	*p*-value		
	CR300	CR600	CR300	CR600		A	B	A × B
Dry matter intake								
Rice straw								
kg/day	3.41	3.41	3.53	3.31	0.08	0.94	0.19	0.20
g/kg BW^0.75^	51.89	52.07	53.69	50.43	0.95	0.93	0.13	0.09
Concentrate								
kg/day	2.38	2.37	2.36	2.35	0.06	0.70	0.83	0.93
g/kg BW^0.75^	36.18	36.05	35.84	35.82	0.22	0.22	0.73	0.81
Fresh cassava root								
kg/day	1.16	2.31	1.16	2.31	–	–	–	–
g/kg BW^0.75^	17.56	35.29	17.58	35.25	–	–	–	–
Total intake								
kg/day	6.95	8.09	7.04	7.97	0.13	0.89	< 0.01	0.42
g/kg BW^0.75^	105.62	123.47	107.11	121.50	1.05	0.82	< 0.01	0.13
Nutrient intake, kg/day								
Organic matter	5.48	5.91	5.56	5.79	0.08	0.83	< 0.01	0.19
Crude protein	0.39	0.41	0.37	0.39	0.43	0.11	0.12	0.37
Non-fiber carbohydrate	2.69	3.53	2.77	3.56	0.09	0.50	0.03	0.29
Neutral detergent fiber	3.59	4.42	3.62	4.13	0.10	0.22	< 0.01	0.14
Acid detergent fiber	2.58	3.13	2.63	3.06	0.04	0.78	< 0.01	0.20
Nutrient digestibility, %								
Dry matter	65.51	63.71	63.41	59.87	3.00	0.34	0.39	0.78
Organic matter	70.90	71.55	70.81	66.51	2.94	0.40	0.54	0.42
Crude protein	62.66	61.41	59.47	55.29	4.29	0.35	0.61	0.82
Neutral detergent fiber	65.47	72.63	63.52	70.78	2.84	0.52	0.02	0.99
Acid detergent fiber	51.04	54.14	47.44	50.06	4.16	0.37	0.51	0.96

*SEM* standard error of mean.

A is *p*-value level of *Enterococcus faecium*; *E. faecium* (10^8^ CFU/ml) and 3% sulfur. B is *p*-value level of fresh cassava root. CR300: fresh cassava root containing HCN at 300 mg/kg DM/day; CR600: fresh cassava root containing HCN at 600 mg/kg DM/day. Non-fiber carbohydrate (NFC) was calculated according to the equation: NFC (%) = 100 − (CP + NDF + EE + ash).

### Blood profiles and rumen’s parameters

**Figure 1:**
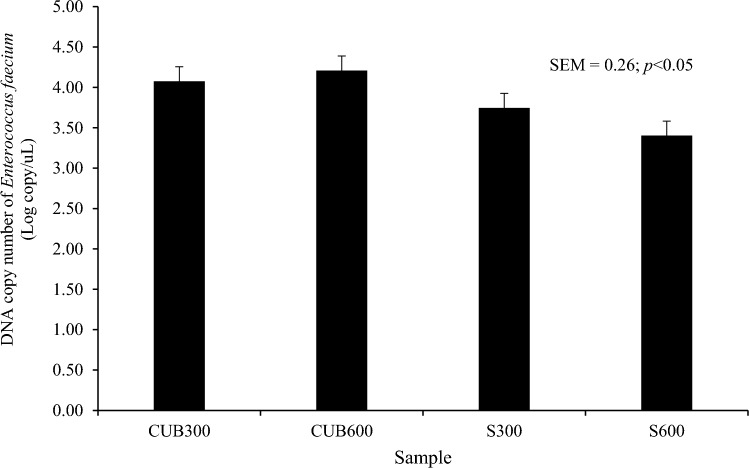
DNA copy of cyanide-utilizing bacteria in the rumen fluid of Thai native beef cattle fed two levels of fresh cassava root (CR) with cyanide-utilizing bacteria and sulfur, CUB300: oral cyanide-utilizing bacteria with fresh cassava root containing HCN at 300 mg/kg DM, CUB600: oral cyanide-utilizing bacteria with fresh cassava root containing HCN at 600 mg/kg DM, S300: concentrate diet containing 3% dry matter of pure sulfur with fresh cassava root containing HCN at 300 mg/kg DM, S600: concentrate diet containing 3% dry matter of pure sulfur with fresh cassava root containing HCN at 600 mg/kg DM].

Table 3 shows the results of CUB and sulfur supplemented with CR containing HCN at 300 mg/kg DM and 600 mg/kg DM, according to the study, prior to feeding and after 4 h of feeding beef cattle. The ruminal pH, microorganism population, NH_3_–N, blood urea-N, and blood thiocyanate did not change when CUB and sulfur were added with two levels of CR (*p* > 0.05). The efficiency of HCN degradation in the rumen varied from 87.26 to 87.54% units regardless of whether CUB or sulfur was added (*p* > 0.05). In contrast to the degradation efficiency seen in cattle fed CR with an HCN content of 300 mg/kg DM, the degradation efficiency of HCN was significantly greater when cattle were provided with CR containing HCN at a concentration of 600 mg/kg DM (p < 0.05). When beef with CUB or sulfur was exposed to 600 mg/kg DM of HCN in CR, the decomposition of HCN was enhanced to 90.11% units. In addition, the bacterial population and HCN concentrations were impacted by CR containing HCN at 300 mg/kg DM and 600 mg/kg DM (*p* < 0.01). The bacterial count increased from 9.66 to 9.83 Log_10_ cells/ml as the CR containing HCN concentration rose from 300 to 600 mg/kg DM. In Fig. 1, a DNA copy of *E. faecium* was found in the rumen fluid of Thai native beef cattle that had been given two levels of CR as well as either CUB or sulfur as an additive. As compared to the group that was given sulfur, it was discovered that the population of *E. faecium* was significantly higher in the group that was fed CUB (*p* < 0.05). However, when comparing CR levels, no statistically significant variations were observed between concentrations of *E. faecium* (p > 0.05).

**Table 3:** Ruminal fermentation, degradation efficiency of cyanide in the rumen, and blood metabolites of Thai native beef cattle fed two levels of fresh cassava root (CR) with cyanide-utilizing bacteria and sulfur.

Items	*Enterococcus faecium* (10^8^ CFU/ml)		3% Sulfur		SEM	p-value		
	CR300	CR600	CR300	CR600		A	B	A × B
Rumen ecology								
Ruminal pH								
0 h post feeding	6.99	6.94	6.96	6.89	0.06	0.57	0.36	0.89
4 h post feeding	6.91	6.70	6.83	6.79	0.07	0.97	0.13	0.27
Ruminal microbes, cell/ml								
Bacteria								
0 h post feeding	9.67	9.68	9.66	9.81	0.04	0.21	0.10	0.14
4 h post feeding	9.68	9.78	9.64	9.88	0.04	0.52	< 0.01	0.17
Protozoa								
0 h post feeding	9.09	9.24	9.17	9.26	0.07	0.52	0.12	0.71
4 h post feeding	9.17	9.27	9.23	9.25	0.04	0.66	0.20	0.41
Fungi								
0 h post feeding	8.66	8.70	8.66	8.69	0.04	0.97	0.36	0.97
4 h post feeding	8.78	8.86	8.75	8.80	0.04	0.30	0.12	0.69
NH_3_-N concentration, mg/dl								
0 h post feeding	15.98	16.12	15.63	15.40	0.98	0.59	0.96	0.86
4 h post feeding	17.17	17.45	18.19	18.93	0.70	0.10	0.49	0.75
Degradation efficiency of cyanide in the rumen (%)								
0 h post feeding	85.66	90.36	84.84	90.54	2.38	0.92	0.04	0.81
4 h post feeding	84.24	89.90	84.06	89.63	0.57	0.53	< 0.01	0.75
Blood urea-N concentration, mg/dl								
0 h post feeding	11.25	10.50	10.50	10.50	0.17	0.21	0.98	0.44
4 h post feeding	11.50	11.25	11.00	10.75	0.94	0.60	0.79	1.00
Blood thiocyanate concentration, mg/dl	4.15	4.37	3.63	4.67	0.60	0.85	0.31	0.51

*SEM* standard error of mean.

A is *p*-value level of *Enterococcus faecium*; *E. faecium* (10^8^ CFU/ml) and 3% sulfur. B is *p*-value level of fresh cassava root. CR300: fresh cassava root containing HCN at 300 mg/kg DM/day; CR600: fresh cassava root containing HCN at 600 mg/kg DM/day.

### Concentration of volatile fatty acids (VFAs)

Table 4 shows the effects of CUB and sulfur supplementation with CR on total volatile fatty acid concentration and VFA profiles. Cyanide-utilizing bacteria or sulfur supplementation with CR had no interaction effect between total VFA and their profiles (*p* > 0.05). Nevertheless, the inclusion of CR with an HCN content of 600 mg/kg DM resulted in a significantly higher concentration of propionic acid at both 0 h and 4 h after feeding, as compared to the inclusion of CR with an HCN content of 300 mg/kg DM (*p* < 0.05).

**Table 4:** Concentration of volatile fatty acid of Thai native beef cattle fed two levels of fresh cassava root (CR) with cyanide-utilizing bacteria and sulfur.

Items	*Enterococcus faecium* (10^8^ CFU/ml)		3% Sulfur		SEM	p-value		
	CR300	CR600	CR300	CR600		A	B	A × B
Total VFA, mmol/l								
0 h post feeding	175.65	185.67	177.72	185.63	5.20	0.85	0.11	0.84
4 h post feeding	200.04	202.99	199.95	201.56	1.26	0.56	0.96	0.61
VFA profiles, %								
Acetic acid								
0 h post feeding	82.94	81.42	73.65	75.60	3.06	0.16	0.96	0.71
4 h post feeding	86.95	88.10	80.52	78.97	3.25	0.12	0.96	0.76
Propionic acid								
0 h post feeding	15.56	18.56	14.40	17.41	1.14	0.33	0.02	0.99
4 h post feeding	17.89	21.05	17.50	19.81	1.20	0.51	0.04	0.73
Butyric acid								
0 h post feeding	4.91	5.62	4.63	6.22	0.50	0.54	0.21	1.00
4 h post feeding	6.44	6.45	5.80	6.47	0.42	0.51	0.56	0.57
Acetate to propionate ratio								
0 h post feeding	5.33	4.39	5.11	4.34	0.77	0.91	0.23	0.24
4 h post feeding	4.86	4.19	4.60	3.99	0.69	0.08	0.45	0.20

*SEM* standard error of mean.

A is *p*-value level of *Enterococcus faecium*; *E. faecium* (10^8^ CFU/ml) and 3% sulfur. B is *p*-value level of fresh cassava root. CR 300 mg/kg DM: fresh cassava root containing HCN at 300 mg/kg DM/day; CR 600 mg/kg DM: fresh cassava root containing HCN at 600 mg/kg DM/day.

### Nitrogen utilization

The results of adding CUB and sulfur to CR containing HCN at 300 mg/kg DM and 600 mg/kg DM are shown in Table 5. No interaction effects on nitrogen intake, excretion, or utilization were observed in Thai native beef cattle (*p* > 0.05). Nitrogen intake was affected by the addition of CUB or sulfur and the level of fresh cassava. However, there were no significant differences seen in the amounts of nitrogen absorption and nitrogen retention across the treatments (p > 0.05).

**Table 5:** Effect of different levels of fresh cassava root on feed intake and N utilization of Thai native beef cattle fed two levels of fresh cassava root (CR) with cyanide-utilizing bacteria and sulfur.

Items	*Enterococcus faecium* (10^8^ CFU/ml)		3% Sulfur		SEM	*p*-value		
	CR300	CR600	CR300	CR600		A	B	A × B
Nitrogen (N) balance, g/day								
N intake	61.82	66.06	59.79	63.12	0.33	< 0.01	< 0.01	0.20
N faecal	29.27	32.12	30.27	33.95	1.81	0.64	0.26	0.70
N urine	4.13	4.17	3.46	5.12	0.87	0.87	0.35	0.37
N absorption	32.55	33.94	30.67	32.57	1.73	0.37	0.36	0.88
N retention	28.42	29.77	27.21	27.45	1.87	0.36	0.68	0.77
N retention to N intake	0.46	0.45	0.46	0.44	0.03	0.68	0.55	0.81

N absorption (g) = N intake (g) − N faecal (g). N retention = N absorption (g) − N urine (g). CR300: fresh cassava root containing HCN at 300 mg/kg DM/day; CR600: fresh cassava root containing HCN at 600 mg/kg DM/day.

*SEM* standard error of mean.

A is *p*-value level of *Enterococcus faecium*; *E. faecium* (10^8^ CFU/ml) and 3% sulfur. B is *p*-value level of fresh cassava root.

## Discussion

The animals consumed a high CR level, resulting in a high feed intake. When CR containing HCN at 600 mg/kg DM was supplemented, total intake increased by 14.8% when compared to CR containing HCN at 300 mg/kg DM. In agreement, Supapong et al.^31^ discovered that when CR levels increased, it also increased the total amount of feed that was consumed by the animals. Fresh cassava roots are typically more palatable and offer a more appealing taste and texture compared to the dried form, resulting in increased feed intake^4,6^. Furthermore, due to its high non-fiber carbohydrate (NFC) content, the group supplemented with CR containing 600 mg/kg DM of HCN exhibited a higher nutrient intake value compared to the group supplemented with 300 mg/kg DM of HCN in CR. Non-fiber carbohydrates are a primary source of readily available energy in animal diets. These carbohydrates include sugars and starches, which are more energy-dense than fiber. When animals consume feed with a high NFC content, they can rapidly acquire a significant amount of energy. The improved palatability of the feed can stimulate their appetite, encouraging them to consume more of the NFC-rich feed^4,6,31^. As a result, nutritional intake varies depending on how much CR was provided. The digestibility of NDF varied with the amount of CR fed, with the highest levels found in the group receiving HCN 600 mg/kg DM. When the amount of soluble carbohydrates was increased, the rumen bacteria increased^32,33^. Furthermore, rhodanese and mercaptopyruvate sulfurtransferase, two enzymes that promote HCN conversion, can be used to supply HCN as a nitrogen source for microbial production^14^. The rumen environment would benefit from the addition of nitrogen supply with fermentable starch from cassava, which would also improve microbial growth on digestibility^34^.

The efficiency of HCN degradation in the rumen was about 80% unit, regardless of the addition of sulfur or CUB. These results suggest that CUB or sulfur can potentially reduce HCN from CR and can be added to ruminant feed without endangering the welfare of the animals. HCN degradation efficiency increased by 5.41% when beef was given HCN at 600 mg/kg DM as opposed to 300 mg/kg DM. It’s probable that HCN might serve as a readily available supply of nitrogen for microbiological cell synthesis, in particular for the bacteria responsible for degrading HCN^6,13^. Furthermore, it was discovered that introducing high amounts of HCN led to high degrading efficiency and that either CUB or sulfur might be more effective^5^.

Blood urea nitrogen serves as a biomarker of nitrogen metabolism and has a strong correlation with rumen ammonia levels. There was no significant change in BUN concentration among the different treatments. Blood urea nitrogen levels varied from 10.75 to 11.50 mg/dl when blood samples were taken 4 h after consumption. This phenomenon may be attributed to the consumption of an isonitrogenous concentrate diet by animals, which subsequently leads to no significant alteration in BUN levels or ruminal ammonia concentration^6^. In addition, a high level of HCN in CR is not a danger for steer consumption, as it does not negatively impact the health status of the steers. This could be attributed to the presence of CUB or efficient sulfur degradation of HCN, which facilitates nitrogen supply for rumen microbial growth.

The content of thiocyanate in the blood serves as an indication for the conversion of HCN through the enzymatic activity of rhodanese in the liver^12^. The presence of a high concentration of HCN can lead to an increase in the production of the enzyme rhodanase in the liver. This enzyme facilitates the synthesis of thiocyanate, which can then be eliminated from the body by urine excretion^13^. However, the current study observed that animals consuming a high quantity of HCN did not have a significant influence on their blood thiocyanate levels, which varied from 3.63 to 4.67 mg/dl. It is possible to elucidate that the introduction of a high dosage of HCN to animals can result in the efficient degradation of HCN by up to 84.06 to 89.90% unit by the use of CUB or sulfur groups. The degradation of HCN might potentially function as a source of nitrogen and supply for the growth of rumen bacteria^14^. Hence, the presence of a low HCN level in the rumen may indicate a limited availability of substrates for thiocyanate synthesis in the bloodstream. Consequently, the outcomes of all animal tests demonstrate no discernible differences, thereby ensuring that HCN derived from fresh cassava roots does not pose any risk to animals.

Furthermore, CR containing HCN at 300 and 600 mg/kg DM influenced the bacterial population and HCN concentrations. The bacterial count increased to 9.83 Log10 cells/ml as the CR concentration increased to 600 mg/kg DM. Similarly, Prachumchai et al.^4^ found that at 4 h after feeding, maximum CR supplementation at 20 g/kg BW in combination with pellets containing high sulfur (PELFUR) at 30 g/kg had the largest bacterial population. According to Cherdthong et al.^6^, bacterial populations increased when CR was added to the base diet, along with block feeding at sulfur levels of 4.0%. The degree of CR affects the degradation efficiency of HCN. In the rumen fluid of Thai native beef cattle that had received two amounts of CR as well as either CUB or sulfur as an additive, a DNA copy of CUB was altered. The population of CUB was found to be significantly higher in the group that was fed CUB compared to the group that received sulfur. It seems possible that the large number of CUB in the CUB-fed group was related to the high quantity of CUB supplementation given to the cattle^18^. The DNA copy of *E. faecium* was determined in the rumen fluid of cattle that fed fresh cassava root, as it was first verified to be the predominant population of cyanide-utilizing bacteria in the rumen^17^. The population of *E. faecium* exhibited its maximum levels in the group of animals that had oral inoculation with cyanide-utilizing bacteria, as opposed to the group that was fed sulfur. The potential positive impacts of the *E. faecium* KKU-BF7 inoculants on cyanide may be attributed to their rhodanese activities in the process of cyanide detoxification as well as their ability to break down glycosides as a nitrogen source^12,14^. By utilizing fresh cassava root as a source of carbohydrates and incorporating nitrogen, *E. faecium* can enhance cell proliferation compared to a group with limited nitrogen availability^17,18^. In addition, supplementation of *E. faecium* KKU-BF7 into the rumen by oral inoculant might be another reason for the increasing population of *E. faecium*. This finding demonstrated that CUB was successfully served up in the rumen following oral inoculant administration and also indicated that the treatment had the potential to reduce HCN concentrations.

Fresh cassava root containing HCN at 600 mg/kg DM resulted in higher propionic acid concentrations than HCN at 300 mg/kg DM. Due to the fact that CR contains NFC, which the rumen can easily convert into propionate, beef cattle that were given a higher dose of CR showed higher ruminal propionate concentrations^32,33^. Supapong et al.^31^ discovered that giving animals more CR led to increased amounts of propionic acid after 4 h of feeding. Like with Cherdthong et al.^6^, who showed that beef cattle given CR at 1.5% BW may have a 15.4% increase in propionate.

The addition of CUB or sulfur, as well as the amount of fresh cassava, influenced nitrogen intake. The group that received CUB supplements consumed more nitrogen per day than the group that received sulfur supplements. This could be because CUB can use HCN from CR to generate nitrogen for their growth, resulting in more digested feed consumption^17^. As a result, the intake of nutrients, particularly N, has increased. Furthermore, when CR supplementation was increased, the cattle’s daily nitrogen intake increased by 4 g/day. This could be because the animal received more N in the CR, resulting in increased N intake. Supapong et al.^31^ found that adding CR increased nitrogen availability in native beef cattle from 33.5 to 40.0 g/day.

## Conclusions

Based on the experiment’s findings, there were no significant differences observed in feed intake, nutritional digestibility, HCN degradation efficiency, or blood metabolites between the groups with added CUB and sulfur. However, giving CUB orally to cattle increased the *E. faecium* population compared to the sulfur group. Moreover, including HCN at a 600 mg/kg DM concentration in CR can enhance feed intake, NDF digestion, HCN degradation, and propionate levels. While both CUB and sulfur have similar effects, CUB appears more promising due to the potential adverse effects of excessive sulfur intake, such as reduced feed consumption, diarrhea, and muscle spasms in ruminant animals. Therefore, oral CUB administration could be a better alternative for CR usage, offering benefits like HCN detoxification and increased *E. faecium* population. Future research could explore the feasibility of a powdered CUB form for incorporation into concentrated diets, which might be more practical and less stressful for animals than the current oral solution method.

## Data Availability

The datasets used and/or analysed during the current study available from the corresponding author on reasonable request.
